# Myoblasts rely on TAp63 to control basal mitochondria respiration

**DOI:** 10.18632/aging.101668

**Published:** 2018-11-28

**Authors:** Veronica Ciuffoli, Anna Maria Lena, Alessandra Gambacurta, Gerry Melino, Eleonora Candi

**Affiliations:** 1 Department of Experimental Medicine and TOR, University of Rome “Tor Vergata”, Rome, Italy; 2 MRC-Toxicology Unit, University of Cambridge, UK; 3 IDI-IRCCS, Biochemistry Laboratory, Rome, Italy

**Keywords:** p63, myoblasts differentiation, mitochondria, metabolism

## Abstract

p53, with its family members p63 and p73, have been shown to promote myoblast differentiation by regulation of the function of the retinoblastoma protein and by direct activation of p21^Cip/Waf1^ and p57^Kip2^, promoting cell cycle exit. In previous studies, we have demonstrated that the TAp63γ isoform is the only member of the p53 family that accumulates during *in vitro* myoblasts differentiation, and that its silencing led to delay in myotube fusion. To better dissect the role of TAp63γ in myoblast physiology, we have generated both sh-p63 and Tet-On inducible TAp63γ clones. Gene array analysis of sh-p63 C2C7 clones showed a significant modulation of genes involved in proliferation and cellular metabolism. Indeed, we found that sh-p63 C2C7 myoblasts present a higher proliferation rate and that, conversely, TAp63γ ectopic expression decreases myoblasts proliferation, indicating that TAp63γ specifically contributes to myoblasts proliferation, independently of p53 and p73. In addition, sh-p63 cells have a defect in mitochondria respiration highlighted by a reduction in spare respiratory capacity and a decrease in complex I, IV protein levels. These results demonstrated that, beside contributing to cell cycle exit, TAp63γ participates to myoblasts metabolism control.

## Introduction

Myogenesis is an ordered process driven by myogenic regulatory factors, coordinated with permanent cell cycle withdrawal and culminating in myoblasts fusion in myotubes. Myogenic differentiation process requires bHLH transcription factor family of myogenic regulatory factors (MRFs), including MyoD, Myogenin and Mrf4 which lead to expression of muscle-specific proteins, such as myosin heavy chain (MHC) and creatine kinase (CK) and to multinucleated myotube formation [1]. Cell cycle arrest occurs early during the differentiation program playing a key role in myoblast differentiation into mature myotubes [2–8]. In addition to autophagy [9,10], the retinoblastoma protein (Rb) is a key factor, indeed myoblasts lacking Rb fails to exit the cell cycle [11–13]. *In* *vitro* and *in vivo* experiments demonstrated that also the cyclin-dependent-kinase inhibitors p21^Cip/Waf1^ (p21) and p57^Kip2^ (p57) are important during myogenesis, acting redundantly to inhibits cell cycle arrest during myoblast differentiation [10,14–16]. Upregulation of p21 and the dephosphorylation of retinoblastoma protein (pRb) appear to be critical regulatory events for the establishment of both the postmitotic and apoptosis-resistant states, the latter is relevant for controlling muscle mass and thereby the size of individual motor units [17].

Several studies indicated that p53 and its family members, p63 and p73, are also involved in myoblast differentiation [13,18–24], regulating cell cycle exit and the early stage of myogenesis. TP53 is primarily related to its tumour suppression function [25–27] despite solid evidence of a differentiation function [28–31]. TP63 and TP73 genes are transcribed by two different promoters, giving rise to multiple isoforms with own properties, the (TA) isoforms with an N-terminal transactivation domain and the N-terminal truncated (ΔN) isoforms. Moreover alternative splicing at mRNA 3’-end generates isoforms with different C-temini length and sequence (α, β, γ, δ and ε) and also different properties [32–36]. All p53 family members are involved in myoblast differentiation and rhabdomyosarcoma development [13,16]. TP63 is essential for skin development [13,37,38], but plays also a crucial role in cancer biology [39,40] as well as in skeletal muscle homeostasis [41]. Indeed, TAp63γ isoform is the only member of the p53 family that accumulates during *in vitro* myoblasts differentiation and its silencing leads to a delay in terminal differentiation and a reduction of the fusion index [16]. To date the specific role of TAp63γ has not been investigated. Here, using as experimental system the well established *in vitro* model involving the immortalized murine myoblast C2C12 and C2C7 cell lines [42], we asked the question whether TAp63γ has a specific role in myoblasts, independent from the other family member p53 and p73. We generated sh-p63 and Tet-ON doxycycline-inducible TAp63γ clones to study the effects of TAp63γ depletion and/or ectopic expression in proliferating myoblasts. By whole transcriptome mRNA profiling, we found that p63-depletion affects the expression of genes involved in proliferation and metabolism. Indeed, sh-p63 knock-down cells present an increased cell proliferation rate, while TAp63γ over-expression decreases proliferation. Interestingly, p63 depletion affects also mitochondria functions as indicated by the reduction of the spare respiratory capacity, the decrease in complex I and IV protein levels and ATP reduction. Furthermore, mitochondria reactive oxygen species increase and the NADP/NADPH ratio decrease. Overall, these results indicate that TAp63γ participate to myoblasts proliferation controlling both cell cycle exit and mitochondrial metabolism.

## RESULTS

### TAp63 modulates gene expression in myoblasts

**Figure 1:**
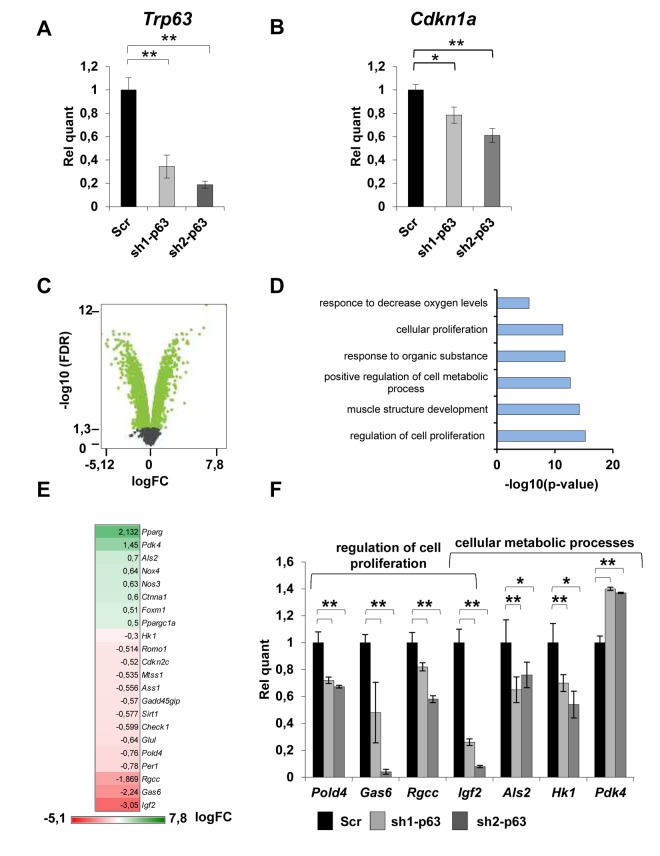
**TAp63 knock-down affects the expression of genes involved in proliferation and metabolism.** (**A**) RT-qPCR of p63 mRNA (Trp63) and (**B**) p21 (Cdkn1a) performed in proliferating Scr, sh1-p63 and sh2-p63 clones. Results are shown as average of three experiments ± s.d. *p<0.05. (**C**) Volcano plot showing -log_10_(FDR) in function of the log_2_(FC) for coding genes in Scr and sh1-p63. Green points indicate significantly expressed genes (**D**) GO terms of microarray performed on significantly modulated genes in C2C7 myoblasts (**C**). Panther was used for biological process (http://pantherdb.org/). (**E**) Heatmap expression level of genes from “*positive regulation of cell proliferation*” and “*regulation of cellular metabolism*” categories. (**F**) RT-qPCR analysis of mRNA level of modulated genes. Results are shown as average of three experiments ± s.d. *p<0.05, **p<0.01.

To investigate the impact of p63 depletion in myoblasts, we performed a whole transcriptome mRNA expression profiling in C2C7 cells. To this aim, we generated stable scramble (Scr) and sh-p63 C2C7 clones (sh1- and sh2-p63) (Fig. 1A). The used sh sequences are designed to knock down all p63 isoforms. As control, we showed that sh1- and sh2-p63 clones showed a reduction of the *bona fide* TAp63 direct target, Cdkn1a (p21) (Fig. 1B). Furthermore, the reduction of TAp63 expression in these clones was maintained also during differentiation as indicated by TAp63 RT-qPCR (Fig. S1A-B). sh1- and sh2-p63 cells also presented a delay in differentiation as evaluated by phase contrast microscopy, immunofluorescence for MyHC and by western blots against MyHC and MyoG proteins (Fig. S1B-D), confirming previous obtained results [16]. By whole transcriptome mRNA profiling, we compared scramble *versus* sh1-p63 transcripts. In this conditions, we have identified 2123 up-regulated and 1989 down-regulated genes (cut-off log(FC)< -0,5 and log(FC)>0,5, p-value<0,05; Fig. S1C, Table S1). Gene Ontology analysis of significantly modulated genes were classified in different groups linked to “muscle structure development”, “regulation of cell proliferation”, “regulation of cell metabolic process” (Fig. 1D). Genes included in the “regulation of cell proliferation” and “regulation of cell metabolic process” categories are indicated in Fig. 1E. Selected genes related to proliferation (Pold4, Gas6, Rgcc, Igf2) and metabolism (Als2, HK1, Pdk4) were validated by independent RT-qPCRs (Fig. 1F**,** 0.001<p<0.05). These results demonstrated that beside regulating genes involved in proliferation, TAp63γ modulates also the expression of genes involved in metabolism.

### TAp63 knock-down affects myoblast proliferation

**Figure 2:**
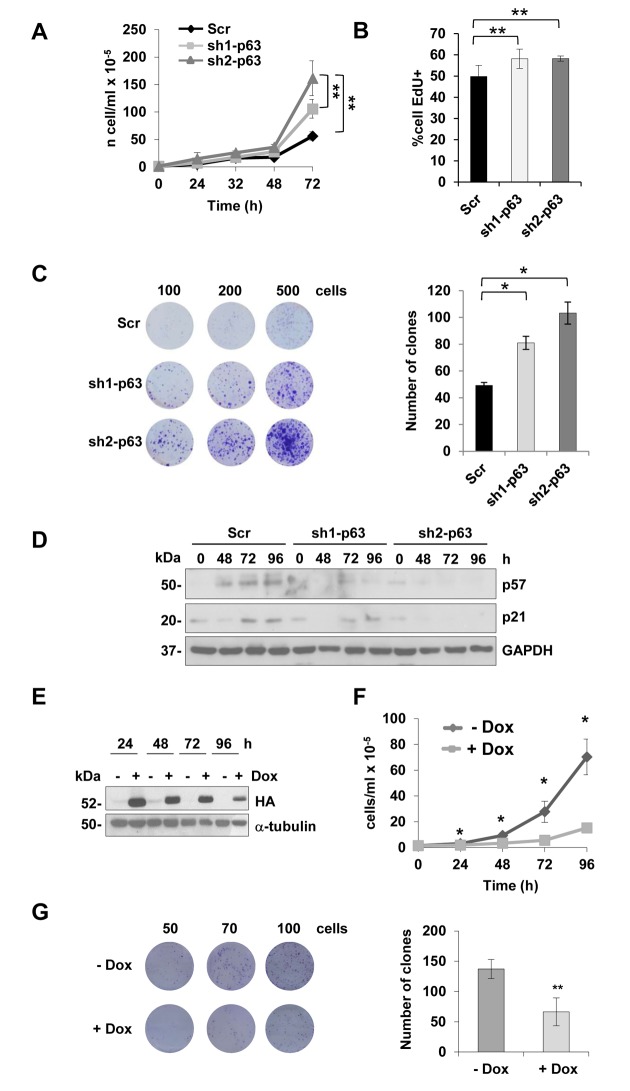
**TAp63 knock-down increases myoblast proliferation.** (**A**) Growth curve of C2C7 Scr, sh1- and sh2-p63 clones. One representative experiment of three is shown. (**B**) EdU-incorporation assay in proliferating C2C7 scramble Scr, sh1- and sh2-p63 clones. Data are shown as mean± S.D. **p<0,01 by T-student test. **(C)** Clonogenicity assay of C2C7 Scr, sh1- and sh2-p63 clones. Images of one experiment of three are shown*.* Colony number count/dish is reported in the histogram (right). *p< 0,05 by T-student test. Data are shown as mean ± S.D. of three independent experiments. (**D**) Western blot confirming reduced expression of p21 and p57 in sh1- and sh2-p63. One representative experiment of three is shown. **(E)** Western blot analysis of C2C12 upon doxycyclin treatments (Tet-ON, Dox). Tubulin is shown as loading control. One representative experiment of three is shown. (**F**) Growth curve of Tet-ON TAp63γ C2C12 cells after 24, 48, 72 and 96h of Dox (2µg/ml) induction. (**G**) Clonogenicity assay of Tet-ON TAp63γ C2C12 cells after 6 days doxycycline (2µg/ml) induction. Colony number count/dish is reported in the histogram *(right).* Data are shown as mean ± S.D. of three independent experiments. *p< 0,05; **p< 0,01.

To better characterize the role of p63 depletion in myoblasts, we evaluated cell proliferation in sh1- and sh2-p63 C2C7 clones. Growth curves showed a significant increase of cell number at 72 hours (Fig. 2A, p<0,01). This was confirmed evaluating cells in S-phase by EdU-incorporation assay (Fig. 2B, p<0.01) and by clonogenic assay, in which the clone numbers increased 1.5 and 2.2 fold over scramble cells for sh1- and sh2-p63 clones, respectively (Fig. 2C, p<0.05). Time courses evaluating p57 and p21 expression by western blots (Fig. 2D), are in line with enhanced proliferation capacity of the sh1- and sh2-p63 myoblasts, in which p21 and p57 expression decreased. We generated an additional cellular model to further confirm these findings using the Tet-ON system. C2C12 myoblasts expressing TAp63γ under the control of doxycycline (Dox) were used to demonstrate the opposite; indeed doxycycline addition resulted in a decrease of cells number, that is evident already after 24 hours after induction (Fig. 2E-F, p<0.05) and confirmed in shorted time-points (8, 16 and 24 hours of induction) by cell cycle analysis (Fig. S2A-B). As control, we confirmed that induction of TAp63γ does not induce cell death in these cells, evaluated as hypo-diploid events (Sub-G1 events, Fig. S2C). Clonogenic assays also confirmed the strong TAp63γ anti-proliferative effect, resulting in reduction of about 50% of the clone (Fig. 2G, p<0.05). Altogether these data indicated that, *in vitro*, TAp63γ is involved in controlling myoblast proliferation.

### TAp63 knock-down induces mitochondrial-derived oxidative stress

**Figure 3:**
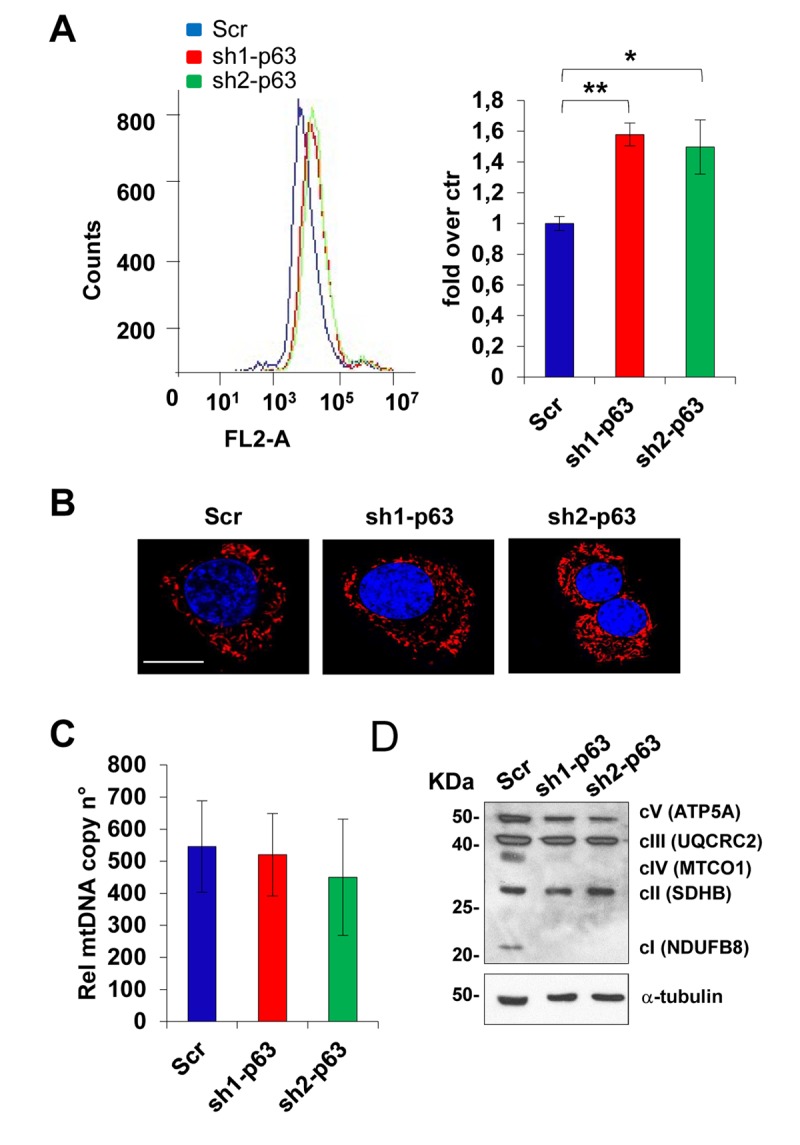
**TAp63 knock-down affect mitochondrial functions.** (**A**) Mitosox Red analysis by flow cytometry in C2C7 Scr, Sh1 and Sh2 clones to assay mitochondrial superoxide anion levels. One of three independent experiments is shown *(left)*. Quantification of fluorescence intensity is shown in the histogram *(right)*. Data are shown as mean ± S.D. from three independent experiments. *p< 0,05 and **p< 0,01 by T-student test. (**B**) Mitotracker Red staining to detect mitochondrial content and shape. One representative experiment is shown. Magnification bar: 10μm. (**C**) qPCR relative to mitochondrial DNA copy number quantificated by the expression level of mitochondrial gene *Nd5* and *12S* related to expression of single copy gene *Sdha*. One from three independent experiments is shown. (**D**) OXPHOS antibody mixture was used to detect mitochondrial protein level by western blot in C2C7 scramble control (Scr), sh1- and sh2-p63 clones. One of three independent experiments is shown.

Since p63 depletion has been associated in different cell types to oxidative stress [43–45] in standard culturing conditions, we investigated mitochondrial functions, since they play an important role in oxidative metabolism and represent the cellular source of reactive oxygen species (ROS), as well as in several other essential physiological functions [46–53]. We measured superoxide anion levels production by Mitosox Red analysis by flow cytometry in C2C7 scramble and sh1- and sh2-p63 C2C7 cells. Depletion of p63 leads to an increase in mitochondrial oxidative stress, more specifically O_2_^-^, from 1 to 1.5 fold over control for both sh1- and sh2-p63 cells, respectively (Fig. 3A, p<0.01 and p<0.05), indicating that electrons can escape between complexes and become trapped in oxygen. Though, Mitotracker Red staining, to detect mitochondrial content and shape, did not show differences among Scr and both sh1- and sh2-p63 cells, respectively (Fig. 3B). Furthermore, mitochondrial DNA copy numbers did not change, as evaluated by measuring by RT-qPCR the expression of the mitochondrial genes *Nd5* and *12S* related to the expression of the single copy nuclear gene *Sdha* (Fig. 3C). In an attempt to understand the reason for the O_2_^-^ increases upon p63 depletion, we evaluated by western blot the relative levels of mitochondrial OXPHOS (complexes I–V, Fig. 3D). Results indicate down-regulation of complex IV (MTCO1) and complex I (NDUFB8) proteins upon p63 depletion. These results suggest a link of TAp63 in controlling mitochondrial function in C2C7 myoblasts.

### TAp63 knock-down affects mitochondrial respiration

**Figure 4:**
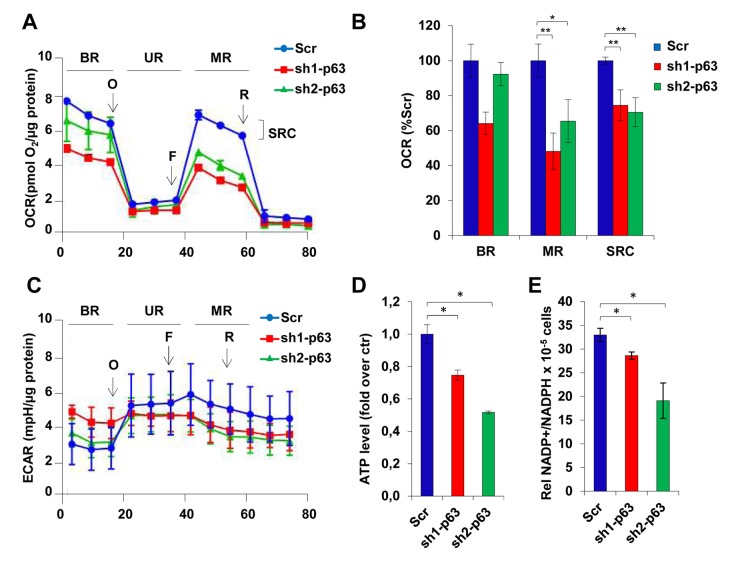
**TAp63 knock-down affects mitochondrial respiration.** (**A**) OCR performed in 6 well seahorse assay plates shows the cellular respiration profile in C2C7 Scr, Sh-1 and Sh2-p63 clones after treatment with the drugs oligomycin (40µg/µl), FCCP (50nM) and rotenone (25nM). One representative of three independent experiments is shown. (**B**) The relative quantification of the area below the curves corresponding to stage BR, UR, MR and SRC (basal respiration, uncoupled respiration, maximal respiration and spare respiratory capacity) is shown in histogram and reported as percentage of Scr. Data are shown as mean ± S.D. of three measures detected after drugs injection and normalized to µg of proteins *p< 0,05 and **p< 0,01. (**C**) ECAR performed in 6 well seahorse assay plates shows the cellular respiration profile in C2C7 Scr, sh1- and sh2-p63 clones after treatment with the drugs oligomycin (40µg/µl), FCCP (50nM) and rotenone (25nM). One representative of three independent experiments is shown. (**D**) ATP levels in C2C7 Scr, sh1- and sh2-p63 clones are normalized to the cell number and are reported as relative quantification to the Scr. Data are shown as mean ± S.D. from three independents experiments *p< 0,05 by Student T-test. (**E**) NADP+/NADPH ratio in Scr, sh1- and sh2-p63 C2C7 clones are normalized to the cell number. Data are shown as mean ± S.D. from three independent experiments *p<0,05; **p<0,01.

Keeping in mind that p53 is known to regulate oxygen consumption with different pathways, as well as its family members TAp73 and ΔNp63 [44,45,54,55], we decided to evaluate aerobic respiration in scramble and p63-depleted cells. We found that p63 depleted cells (sh1-and sh2-p63 clones) had an attenuated mitochondrial respiration with a significantly reduced oxygen consumption rate (OCR, Fig. 4A). Basal mitochondrial respiration is slightly changed between scramble control cells and p63-depleted clones as estimated by measuring OCR (pmol/min). Both the maximal respiratory capacity (MR) and the spare respiratory capacity (SRC) are significantly attenuated in the p63-depleted clones compared to scramble control cells; supporting the idea that mitochondria retain sensitivity to environmental stress (Fig. 4B). Yet, depletion of p63 resulted in the inability of C2C7 to relay on aerobic respiration. Oligomycin injection induced aerobic metabolism in scramble control cells, as underlined by the tendency to increase extracellular acidification rate (ECAR, Fig. 4C), while p63 depleted cells did not responded at all, failing to rescue the cells that were unable to reply on the glycolytic pathway. As matter of fact, ATP level strongly decrease in p63 depleted clones (23% and 45% inhibition in sh1- and sh2-p63 cells, respectively; Fig. 4D). Finally, decreases in NADP+/NADPH ratio in sh1- and sh2-p63 cells (from 33% in scramble cells to 27% and 19% in sh1- and sh2-p63 cells, respectively, p<0.05; Fig. 4E) suggested that the abnormalities in aerobic and anaerobic respiration direct the metabolism toward other metabolic pathways (ie. pentose phosphate). NADPH increases confirmed also the need of p63-depleted cells to maintain thigh the level of anti-oxidant defence molecules.

## DISCUSSION

Aging involves a complex set of genetic [56–58] and metabolic [59–65] pathways that engage all organs, including muscles [66,67]. In these pathways a role for p53 [68–72] and its family members [55,73] is well established. In particular, p63 is involved in senescence and aging [74,75]. The p63 gene, the ancestral member of the p53 family [32,55,76] clearly involved in cancer [77–80], is crucial for the development as well as for the adult homeostasis of the epidermis [34,81]. Here, through its transcriptional activity, it regulates the early differentiation and formation of the cornified envelope [82,83] as well as the apoptotic [84], senescence [85,86] and metabolic [36,44,87] activities of the skin. This function is directly and indirectly related to the barrier function of the skin, with its complex pathways [82,88–90] intended also at the immunological and inflammatory protection of the organism [91–95] and thus preventing unwanted immunopathologies [96–101]. Nonetheless, recent findings suggest also an additional role in myogenesis [16,19].

Several studies investigated the role of p53 and its family members in myoblasts differentiation, showing that while p53 directly regulated protein level of the retinoblastoma (RB) protein, p63 and p73 cooperates to RB activation, *via* their target gene cyclin kinase inhibitor p57^KIP2^, that maintains RB in an active hypo-phosphorylated state [13]. Previous studies generated in our laboratory point out at specific role of TAp63, specifically the TAp63γ isoform, during myoblast differentiation. Indeed, among the p53 family members the TAp63γ is the only one that accumulates during differentiation and knock-down of TAp63γ strongly delays and impairs myoblasts differentiation, as indicated by the reduction of specific markers and of the mitotic fusion index [16]. Here, we confirmed, using as model both sh-p63 C2C7 myoblasts and TAp63γ Tet-ON C2C12 myoblasts, the importance of TAp63γ in controlling cell cycle exit and proliferation. As matter of fact, sh-p63 clones present down-regulation of both p21 (CDKN1A) and p57 (CDKN1C) cyclin-dependent kinase inhibitors. Intriguingly, we found that genes usually expressed in proliferating cells (Gas6, Pold4 and Igf2) were down-regulated in p63-depleted myoblasts, indicating that additional pathways or compensatory mechanisms are engaged to limit cell proliferation in absence of p63. To find additional functions of TAp63γ in myoblasts we performed a gene array, comparing control C2C7 cells with p63-depleted C2C7 myoblasts. Our results demonstrated that in myoblasts lacking TAp63γ many genes related to metabolism are modulated. Furthermore, mitochondria activity appear altered as indicated by the increase of reactive oxygen species of mitochondrial origin and by alteration of the mitochondria function and respiratory capacities. Mitochondria are key organelles to provide, via oxidative phosphorylation, the energy necessary for cellular functions [102–107]. Several experimental evidences indicated that, in different cell types including myoblasts, mitochondria activity is strongly associated to cell differentiation [108–112]. Mitochondria function and activity is finely regulated during differentiation processes, including myogenesis, that was shown to be impaired in respiratory-deficient myoblasts [113]. Mitochondria play an important role also during myoblast proliferation [109,114]. In L6E9 muscle cells, for instance, the increase of mitochondrial oxidative metabolism by pyruvate blocks cells proliferation in G1 and S phases [115]. These data suggest that an oxidative metabolism and mitochondrial biogenesis are often associated to muscle cell differentiation, instead a glycolytic metabolism is required for myoblasts proliferation. Yet, in a different cellular system, it has been shown that p63, by directly controlling the expression of the mitochondrial-associated exokinase II enzyme, favours the coupling between glycolysis and oxidative metabolism [44], suggesting that also in myoblasts p63 could affect directly and/or indirectly both metabolisms. Interestingly, mitochondrial activity is altered in a rat Rhabdomyosarcoma cell line [116]. Rhabdomyosarcoma is the most common soft tissue sarcoma in childhood and adolescents; it arises from skeletal muscle that maintains myoblasts in a proliferative state [117]. As a matter of a fact, RH1 cell line shows a decrease in either ATP level, mitochondrial spare respiratory capacity [116] and a downregulation in CI [118], similarly to the sh-p63 depleted cells. Interestingly, deficiencies of the I-IV complexes expression have been reported in patients affected by several myopathies and neuromuscular diseases [119–123]. Furthermore, defects in mitochondrial ATP synthesis and complex I insufficiency have been also observed in isolated mitochondria from the diaphragm and tibialis anterior of 12 week-old dystrophin-deficient mdx mice [124]. These suggested to us that TAp63 expression and/or activity could be impaired in these pathologies, in keeping with the metabolic alterations observed in aging [125–130].

Overall, in line with the metabolic control in aging [104,131–133], these data indicate that, beside its anti-proliferative role, TAp63, controlling different sub-sets of target genes, may act in myoblasts modulating also mitochondria metabolism to provide the right metabolic platform necessary to allow myoblasts differentiation. These data expand the current knowledge about the involvement of p53 family members in muscle differentiation process, demonstrating that the function of the individual members, such as TAp63, are distinct from the other members. Further investigations will be necessary to address a possible role of TAp63 and its target genes in human myopathies.

## MATERIALS AND METHODS

### Flow cytometer analysis

EdU incorporation during DNA synthesis was evaluated using the Click-it EdU flow cytometer assay according to manufacturer protocol. 10000 events were acquired using BD Accuri C6 Flow Cytometer and cell cycle was analyzed using BD Accuri C6 Software (BD Biosciences).

Tet-ON Tap63γ myoblasts were grown in presence or absence of doxycyclin (2µg/µl) for 8, 16 and 24 hours, fixed 30 minutes at 4 °C in methanol:aceton (4:1), incubated 20 minutes at 37̊C with RNAse A (20µg/ml) (Sigma, USA) and 20 minutes at room temperature with PI (50µg/ml) (Sigma, USA). 10000 events were acquired using BD FacsCalibur, cell cycle phases and subG1 populations were evaluated by ModFit LT^TM^ software (BD Biosciences).

For the detection of mitochondrial anion superoxide, Scr, sh1-p63 and sh2-p63 cells were incubated with MitoSox Red dye (5µM, Invitrogen) for 10 minutes at 37°C and 10000 events were acquired using BD Accuri C6 Flow Cytometer and analyzed using BD Accuri C6 Software (BD Biosciences).

### Cell lines, transfection and lentiviral infection

C2C7 and C2C12 myoblasts [42] were grown in D-MEM supplemented with 20% fetal bovine serum (FBS) or 10% FBS respectively and penicillin-streptomycin (100 U/ml). Myoblast were differentiated switching them in differentiation medium D-MEM supplemented with 2% horse serum and penicillin-streptomycin (100 U/ml).

C2C12 Tet-ON myoblast were transfected with pTRE-HA-TAp63γ construct by Lipofectamine 2000 reagent (Invitrogen) following manufacturer’s protocol. HA-TAp63γ inducible C2C12 (Tet-ON TAp63γ) clones were selected adding hygromycin (800µg/ml) to culture medium. TAp63γ expression was induced by adding doxycyclin to culture medium (2µg/µl). Sh sequences used to generate sh-p63 stable clones were designed to knock-down all p63 isoforms. Clones were generated as previously described [16].

### Growth curve and clonogenicity assay

Scr, sh1-p63 and sh2-p63 clones growth curves were obtained by counting cells 24, 32, 48, 60 and 72 hours after seeding while Tet-ON Tap63γ C2C12 cells were counted 24, 48, 72, and 96 hours after seeding. Cells were counted by TC20 Automated Cell Counter (BIO-RAD).

For clonogenicity assay 50, 100 and 200 Scr, sh1-p63 and sh2-p63 cells and 50, 70 and 100 Tet-ON TAp63γ cells were seeded. After 6 days cells were washed in PBS, fixed in glutaraldehyde (6.0% v/v) and stained by crystal violet (0.5% w/v). Colonies were counted after staining.

### Western blotting

Myoblasts were lysed in RIPA buffer (50mM Tris-HCl pH 7.4, 150 mM NaCl, 1% NP40, 0.25%Na-deoxycolate, 1mM AEBSF, 1 mM DTT). 15-40 µg of total protein extracts were analysed by SDS PAGE and blotted onto Hybond PVDF membrane (GE Healthcare). Primary antibodies used were: Oxphos cocktail (MitoScience, 1:250), anti-HA (Biolegend, 1:500), anti-α-tubulin (Sigma, 1:10000). After appropriate horseradish peroxidise conjugated secondary antibodies incubation (Bio-Rad), signal detection was performed with ECL chemioluminescence kit (Perkin Elmer).

### RNA-extraction, RT-qPCR, and whole transcriptome mRNA profiling

Total RNA was isolated using RNeasy Mini Kit (Qiagen) following the manufacturer protocol. 1µg of total RNA was used for cDNA synthesis by the GOScript Reverse Transcription System Kit (Promega). mRNAs relative quantification by Real time RT-qPCR was performed using GOTaq Real-Time PCR System (Promega) in Applied Biosystem 7500 Real-Time PCR System (Applied Biosystem). GAPDH was used as housekeeping gene for normalization. Primers used are reported in Table S2. Gene expression was defined from the threshold cycle (C_t_), and relative expression levels were calculated by using the 2^-ΔΔCt^ method.

Whole transcriptome mRNA profiling was performed by Biogazelle (Ghent, Belgium). Briefly 100ng of total RNA was processed using the Quick Amp labeling kit (Agilent), producing Cy3-labelled cRNA. cRNA was hybridized to a SurePrint G3 Mouse GE 8x60K Microarray (Agilent) and microarrays were analyzed using an Agilent microarray scanner and Feature Extraction software. Probe intensities were background subtracted and normalized using quantile normalization. Normalized probe intensities are log2-based. Normalized mRNA expression data and probe annotation are available on request.

### Immunofluorecence and confocal microscopy

Cells were fixed in 4% paraformaldehyde in PBS for 15’ at RT, washed and permeabilized in 0.25% Triton-X-100 in PBS for 5’. Blocking was performed in 5% goat serum in PBS for 1 hour. Incubation with primary antibodies was at 4̊ C for 16h. Primary antibodies used was MHyC (Sigma, MY32, 1:500) and secondary antibody AlexaFluor 568 conjugated anti-mouse (Invitrogen, 1:1000). Nuclei were stained by DAPI (Sigma, 1µg/mL).

For the detection of mitochondrial shape Scr, sh1-p63 and sh2-p63 cells were incubated with Mitotracker dye (50nM, Invitrogen) together with Hoesch 33342 (1μM, Sigma-Aldrich) for nuclei staining, for 30 minutes at 37̊C.

Cell images were obtained by confocal laser microscope NIKON Eclipse Ti using EZ C.1 software (Nikon).

### Seahorse flux analysis

Oxidative phosphorylation and glycolysis flux were analysed by measuring oxygen consumption rate (OCR) and lattic acid release (ECAR) by XF6 XF analyser (Seahorse Bioscience). 8x10^4^ Scr, sh1-p63 and sh2-p63 myoblast were seeded in XF 6 well-plate. Cells were washed with assay medium (DMEM 8.3 g/L, NaCl 30 mM, GlutaMax 2 mM, sodium pyruvate 1 mM, glucose 11.11 mM, phenol red, pH 7.4) and equilibrated at 37̊C (in CO_2_ free atmosphere). Injections of oligomycin 40 µg/ml, FCCP 50 nM, and rotenone 25 nM were set to analyse mitochondria response. Basal Respiration (BR), Maximal Respiration (MR) and Spare Respiratory Capacity (SRC) were quantified for three successive OCR measurement after each drug injection. Data collected were normalized to µg protein.

### mtDNA copy number quantification

Genomic DNA extraction from Scr, sh1-p63 and sh2-p63 myoblasts was performed using Wizard genomic DNA purification kit (Promega) following manufacturer’s protocol. 10 ng of genomic DNA was used to perform mtDNA copy number evaluation by quantification of mitochondrial genes Nd5 and 12S by Real Time qPCR. SDHA nuclear gene was used for mtDNA copy number normalization. Primers used are reported in Table S2.

### ATP assay and NADP+/NADPH assay

ATP was quantified by ATP Bioluminescent Somatic Cell Assay Kit (SIGMA) following manufacturer’s protocol. Light emission was measured using Lumat LB9507 luminometer (EG&GBerhtodl).

Colorimetric NADP/NADPH Assy Kit (Abcam) was used to detect NADP/NADPH ratio according to manufacturer’s protocol.

Details for all the other methods, including cell cultures, transfection, lentiviral infection, growth curve, clonogenicity assay, western blotting, RNA-extraction, RT-qPCR, whole transcriptome mRNA profiling immunofluorescence and confocal microscopy are described in Supporting Information.

### Bioinformatic analysis

For gene ontology analysis, gene ontology consortium software available on-line was used (http://geneontology.org/).

### Statistical analysis

Statistical analysis of grouped values was performed by T Student test. Differences with p<0.05 were considered significant.

## Supplementary Material

Supplementary Figures

Supplementary Table 1

Supplementary Table 2
