# Supplementary material for: Myoblasts rely on TAp63 to control basal mitochondria respiration
Source: Aging (Albany NY). 2018 Nov 28;10(11):3558–73. doi: 10.18632/aging.101668 (PMC6286837; doi:10.18632/aging.101668)
Supplement: Supplementary Figures [file aging-10-101668-s001.pdf]

SUPPLEMENTARY FIGURES

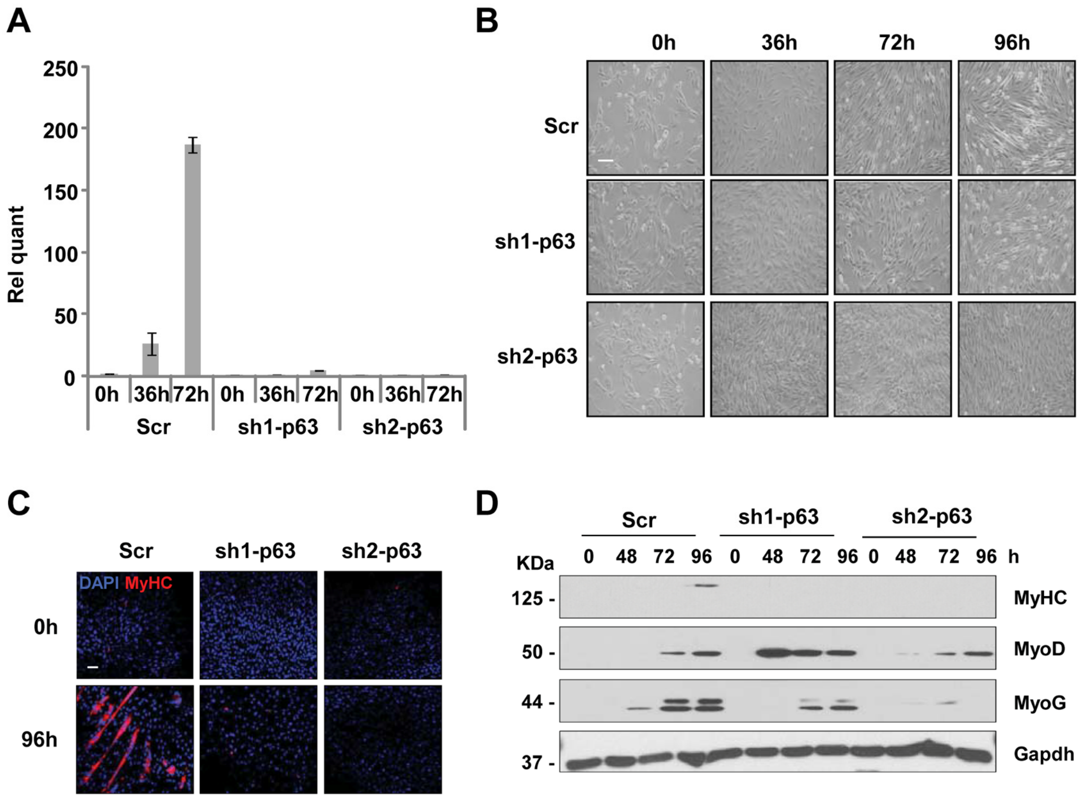

Figure S1. Generation of sh1-p63 and sh2-p63 clones. (**A**) TAp63 mRNA level in control (Scr), sh1- and sh2-p63 C2C7 clones quantified by RT-qPCR. Clones were grown in differentiation conditions for 0, 36 and 72h. (**B**) Phase-contrast microscopy images of proliferating and differentiating Scr and sh1- and sh2-p63 myoblasts. Scale bar: 10µm. (**C**) Immunofluorescence staining for Myosin Heavy Chain (MyHC) at 0 or 96h of differentiation. Scale bar 100µm. One representative experiment of three is shown. (**D**) Western blot analysis of differentiation markers in Scr, sh1- and sh2-p63 C2C7 clones after 0, 48, 72 and 96h of differentiation. One representative experiment of three is shown.

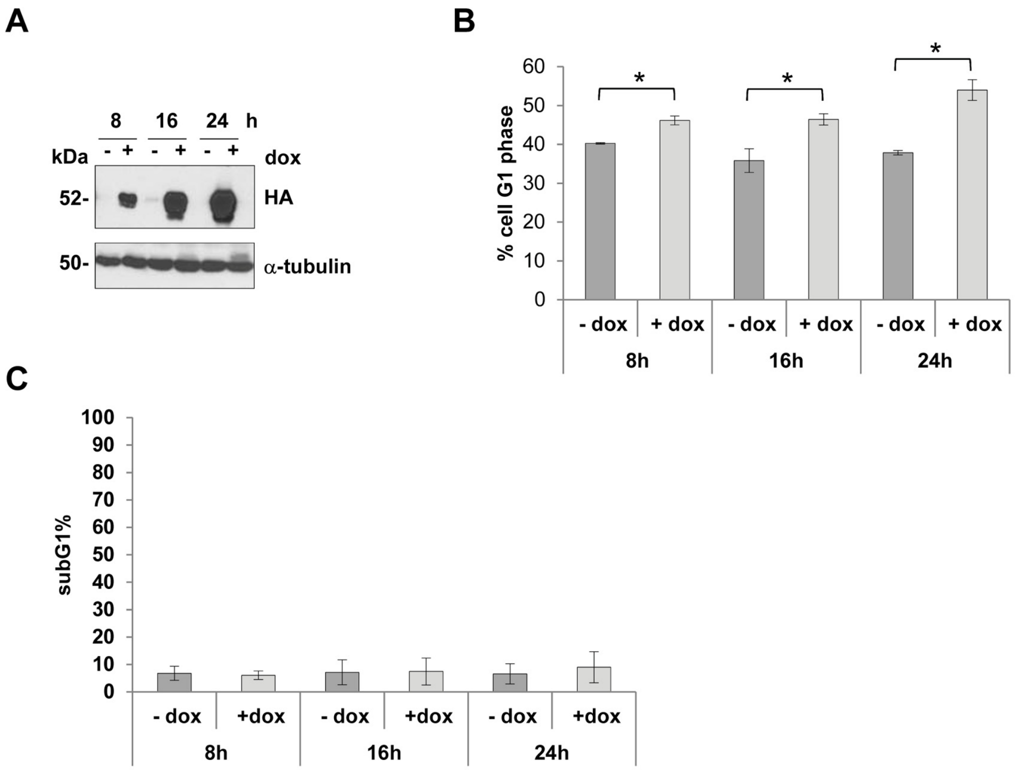

Figure S2. Tet-On inducible TAp63γ expression in C2C12 alters cell cycle. (**A**) TAp63γ overexpression was induced by doxycyclin (2µg/µl) at the indicated time points and detected by western blot analysis using anti-HA antibody. One representative experiment of three is shown. (**B**) Cell cycle evaluation by propidium Iodide (PI) staining and FACS analysis in Tet-On TAp63γ C2C12 cells. (**C**) Evaluation of the subG1 events in cells treated as in (**B**). Data are shown as mean ± S.D. *p<0,05 by T-student test.
