# Supplementary material for: Myoblasts rely on TAp63 to control basal mitochondria respiration
Source: Aging (Albany NY). 2018 Nov 28;10(11):3558–73. doi: 10.18632/aging.101668 (PMC6286837; doi:10.18632/aging.101668)
Supplement: Supplementary Table 2 [file aging-10-101668-s003.pdf]

SUPPLEMENTARY TABLE

Table S2. Primers used in the study.

Genes	Primers
mPold4	F 5’-TCGGAAGCGGTTCATCACTG-3’
	R 5’-GTACAAGGCCCATACTGCCA-3’
mGas6	F 5’-GCTGCAGCTTCGGTACAATG-3’
	R 5’-TGATAGAGGCCCCTCTCCAG-3’
mRgcc	F 5’-TCACTCCTCGGAAAGCCAAA-3’
	R 5’-GTGAACCAAGAATGGCCAGG-3’
mIgf2	F 5’-ATCGTGTTACCACCCAAAGACCCC-3’
	R 5’-GTGATGGAACTGTCCCTGCTCAAG-3’
mAls2	F 5'-GCAGAGTACACACTGAGGTTCT-3'
	R 5'-GGGAACACGTGGTGTGTAGA-3'
mHk1	F 5'-CTGGGTGAGATCGTCCGTT-3'
	R 5'-CGAAGATGCCTCGGGTCT-3'
mPdk4	F 5'-CGCCTGGCCAATATCCCGAA-3'
	R 5'-TCGAACTTTGACCAGCGTGTAAC-3'
mTAp63	F 5’-GCAGAGCACCCAGACAAGCGA-3’
	R 5’-GCACCATTTTCGGAAGGTTCA-3’
mCdkn1a	F 5’-TGTTCCGCACAGGAGCAAAGTG-3’
	R 5’-CGAAGTCAAAGTTCCACCGTTCTCG-3’
mGapdh	F 5’-CCTCGTCCCGTAGACAAAATG-3’
	R 5’-TCTCCACTTTGCCACTGCAA-3’
mt12S	F 5'-CCCAGTTTGGGTCTTAGCTG-3'
	R 5'-ACCGCGGTCATACGATTAAC-3'
mtND5	F 5'-TGATGGTACGGACGAACAGACG-3'
	R 5'-CTGATGCTAGTCATGGGTGGAG-3'
nSdha	F 5'-AGACCTTGAATGAGGCTGAGTGTG-3'
	R 5'-GACACAAAGTGGCGCAACTC-3'
